# Neonatal Lung Disease and Respiratory Failure

**DOI:** 10.1155/2013/238909

**Published:** 2013-03-12

**Authors:** Hercília Guimarães, Anton van Kaam, Gustavo Rocha, Manuel Sánchez Luna

**Affiliations:** ^1^Faculty of Medicine of Porto University, Division of Neonatology of São João Hospital, Porto, Portugal; ^2^Neonatal Intensive Care (H3-228), Emma Children's Hospital AMC, P.O. Box 22660, 1100 DD Amsterdam, The Netherlands; ^3^Division of Neonatology, Hospital de São João, Porto, Portugal; ^4^Paediatrics Complutense University, Neonatology Division, Hospital General Universitario “Gregorio Marañón”, Madrid, Spain

Neonatal lung disease and respiratory failure are common in neonates. Causes of lung disease and respiratory failure are divers and are often associated with maternal pathology, prematurity, and congenital anomalies. Better knowledge and understanding of the pathophysiology of lung disease have led to the development of more effective and safe therapies for both acute and chronic disease.

This special issue includes research articles as well as review articles that will stimulate the continuing efforts to understand the neonatal lung, the pathophysiology of the lung diseases, the development of strategies to treat these conditions, and evaluation of outcomes.

Very preterm infants are commonly exposed to a chronic, often asymptomatic, chorioamnionitis that is usually diagnosed by the histological evaluation of the placenta, only after delivery. G. Rocha extensively reviewed and summarized the available literature on whether histological chorioamnionitis may be associated to lung injury of the preterm newborn. There is a strong evidence that histologic chorioamnionitis is associated with a reduction of incidence and severity of respiratory distress syndrome (RDS). Short-term maturational effects on the lungs of extremely premature infants seem to be, however, accompanied by a greater susceptibility of the lung, eventually contributing to an increased risk of bronchopulmonary dysplasia (BPD). Genetic susceptibility to BPD is an evolving area of research, and several studies have directly related the risk of BPD to genomic variants. There is a substantial heterogeneity across the studies in the magnitude of the association between chorioamnionitis and BPD, and whether or not the association is statistically significant. Recent studies generally seem to confirm the effect of chorioamnionitis on RDS incidence, while no effect on BPD is seen. Recent data have suggested susceptibility for subsequent asthma to be increased on long-term followup. 

S. Gupta and S. M. Donn describe novel approaches to surfactant administration. Surfactant replacement therapy has been the mainstay of treatment for preterm infants with RDS for more than twenty years. Although tracheal instillation is still reputed as the classical method of surfactant delivery, alternative techniques have been investigated. In recent years, the growing interest in noninvasive ventilation has led to novel approaches of administration. These potential strategies include intra-amniotic instillation, pharyngeal instillation, administration via laryngeal mask airway, administration using a thin intratracheal catheter without IPPV, or aerosolized/nebulized surfactant administration in spontaneously breathing infants. Data from clinical trials of these novel techniques will need to evaluate long-term respiratory and neurodevelopmental outcomes and to assess the true cost effectiveness. 

Survival and outcomes for preterm infants with RDS have improved over the past 30 years. F. Flor-de-Lima et al. report the changes in perinatal care and delivery room management at her center in 2005, when early nasal continuous positive airway pressure (NCPAP) and intubate surfactant extubate (INSURE) were introduced, and the positive impact on respiratory outcome and survival of very low birth weight newborns.

M. O'Reilly et al. focus the short- and intermediate-term outcomes of preterm infants receiving positive pressure ventilation in the delivery room. Although recent advances in neonatal care have improved survival rates, rates of BPD remain unchanged. Although neonatologists are increasingly applying gentle ventilation strategies in the neonatal intensive care unit, the same emphasis has not been applied immediately after birth. A lung-protective strategy should start with the first breath to help establish functional residual capacity, facilitate gas exchange, and reduce volutrauma and atelectotrauma. Ideally, a lung-protective strategy should start immediately after birth because the lungs of very preterm infants are uniquely susceptible to injury because they are structurally immature, surfactant deficient, fluid filled, and not supported by a stiff wall. 

Flow-synchronized nasal intermittent positive pressure ventilation (SNIPPV) could be used to reduce endotracheal ventilation, increase successful extubation, decrease the rate of apnea of prematurity, and have better outcome indicated by fewer death and/or BPD in preterm and term newborn infants. C. Gizzi et al. also demonstrate that the introduction of the routine use of SNIPPV after INSURE technique in their NICU reduced the need for MV and favorably affected other short-term morbidities of premature infants <32-week gestation with RDS.

Vascular endothelial growth factor (VEGF), an angiogenic factor secreted by type II pneumocytes, could play a role in congenital diaphragmatic hernia (CDH) pathogenesis. Studies in rodents suggest that VEGF accelerates lung growth in hypoplastic lungs. E. Sanz-López et al. show the changes in the expression of VEGF after fetal tracheal occlusion (TO) in an experimental model of CDH. VEGF protein was significantly lower in fetuses with CDH. TO induced a significant increase in VEGF compared to the fetuses that did not undergo TO.

Patent ductus arteriosus (PDA) is a significant cause of morbidity and mortality in preterm infants. Many factors are associated with closure of ductus arteriosus in preterm infants. K. W. Olsson et al. show that a high ductal flow velocity is associated with successful pharmacological closure of PDA in 22–27-week gestational age infants during pharmacological treatment with cyclooxygenase inhibitors.

O. Carvalho and C. Gonçalves evaluated the lung retinoids content to study the possible difference between male and female mice during prenatal lung development, and to comprehend if the vitamin A metabolism is similar in both genders. They observed that there is a sexual dimorphism in the retinoids content during mice lung development, more evident in the last developmental days, as well as a difference in the retinoids metabolism.

Respiratory syncytial virus (RSV) lower respiratory tract infection is the most common viral respiratory infection in both term and preterm infants. Some studies have been done to determine which risk factors are the best predictors for severe RSV disease. A. Gonçalves et al. evaluated the chest radiographic pattern in RSV disease of the newborn and identified that newborns with a consolidation pattern on admission chest radiograph had a more severe disease course, with greater risk of respiratory support, invasive mechanical ventilation, supplemental oxygen, and prolonged hospitalization.

All of these chapters illustrate some important aspects of the contemporary respiratory neonatal medicine to be adopted in clinical practices and to stimulate experimental or clinical research. 

*Hercília Guimarães* *Anton van Kaam* *Gustavo Rocha* *Manuel Sánchez Luna*

